# Validation of Self-reported Smoking with Urinary Cotinine Levels and Influence of Second-hand Smoke among Conscripts

**DOI:** 10.1038/s41598-017-15526-y

**Published:** 2017-11-13

**Authors:** Yu-Lung Chiu, Shu-Jia Huang, Ching-Huang Lai, Chung-Chi Huang, Shiang-Huei Jiang, Shan-Ru Li, Shu-Ling Hwang, Fu-Gong Lin, Ya-Mei Tzeng, Senyeong Kao

**Affiliations:** 1 School of Public Health, National Defense Medical Center, Taipei, 114 Taiwan; 2 Graduate Institute of Life Science, National Defense Medical Center, Taipei, 114 Taiwan; 3 Center for General Education, National Defense Medical Center, Taipei, 114 Taiwan; 4 Department of Optometry, University of Kang Ning, Taipei, 114 Taiwan

## Abstract

Accurate identification of smoking behaviour is crucial to monitor the smoking rate. This study used urinary cotinine (UC) as a biomarker to verify the effectiveness of self-reported smoking behaviour among conscripts during recruit training. The influence of second-hand smoke (SHS) on the UC concentration was also analysed. A cross-sectional study was conducted from July 2014 to December 2014. The participants comprised a total of 621 military service and basic military training conscripts. A self-administered questionnaire survey and a urine test were performed to verify the participants’ smoking behaviour. The UC concentration of 100 ng/mL was adopted as the baseline to identify smokers. A high level of consistency was observed between the conscripts’ self-reported results and the results validated by the UC concentrations (the overall kappa coefficient was 0.918). Moreover, the overall sensitivity and specificity were 92.9% and 98.1%, respectively. The sensitivity for the military service conscripts was significantly lower than that for the basic military training conscripts (86.1% vs. 97.5%, *P*-value = 0.002). For the self-reported nonsmokers among the military service conscripts, SHS exposure was related to their UC concentrations. The method of self-reporting through a questionnaire survey can serve as a tool to assess conscripts’ smoking behaviour.

## Introduction

Tobacco has been proven to be harmful to human health. Research on tobacco hazards has reported that smoking induces numerous diseases such as chronic lung disease and lung cancer^1^. The general smoking status of various groups should be understood before the implementation of tobacco hazard prevention strategies. Thus, the smoking rate is a crucial indicator for tobacco hazard prevention, which can be determined through the self-reporting of smokers or using biomarker concentrations in those smokers. Cotinine is a major metabolite of nicotine and can be detected in blood, saliva, and urine samples^2,3^. In particular, urinary cotinine (UC) concentration is an easily obtainable and highly effective biological indicator for smoking^4^.

Previous review study have reported that the method of self-reporting, compared with biomarker approaches, may underestimate the smoking rate, and the extent of underestimation varies according to the research participants’ attributes^5^. In Canada, a large, nationwide survey revealed that the self-reported smoking rate of the country was 18.8%; in comparison, the smoking rate determined through the UC concentrations was reported to be 19.1%. Although, the self-reported smoking rate was underestimated, the two results were highly consistent. The sensitivity and specificity of the self-reported smoking rate was 91.6% and 98.3%, respectively^6^. However, studies on specific groups, such as pregnant women and patients with chronic obstructive pulmonary disease, showed that the self-reported smoking rate was significantly lower than that derived using the UC concentrations^7–10^. This might be attributable to the ways in which these groups are prompted to conceal their smoking behaviour under social pressure and expectations, which leads to inaccurate self-reported smoking assessment results.

Throughout the Taiwanese military, a tobacco hazard prevention program, called the National Army Tobacco and Betel Nut Hazard Prevention Project, was initiated jointly by the Ministry of Defense and the Health Promotion Administration in 2003. Specifically, to monitor the smoking rate of the army and the effectiveness of tobacco hazard prevention policies, questionnaire surveys were conducted to estimate smoking prevalence using self-reporting approaches. However, whether such methods are effective in identifying smokers remains to be verified. Furthermore, the effectiveness of tobacco hazard prevention policies can also be monitored by understanding nonsmokers’ level of exposure to second-hand smoke (SHS), and the urinary nicotine metabolite concentrations can be used as an indicator for SHS exposure^11,12^. In Taiwan, conscripts are divided into military service and basic military training conscripts. Military service conscripts are assigned to various units after undergoing two months of recruit training. By comparison, basic military training conscripts are only required to undergo two months of recruit training and two months of specialty training and are not required to continue their military service. In other words, the service time for military service conscripts was longer than that for basic military training conscripts. Conscripts are at the lowest level in the military hierarchy. To meet superiors’ expectations and prevent subsequent controls concerning smoking, conscripts may conceal their smoking behaviour and therefore miss the opportunity to receive adequate assistance in smoking cessation in the military. Few studies have investigated the consistency between the smoking status self-reported by the conscripts and that obtained by measuring the UC concentrations. Therefore, this study aimed to evaluate the prevalence of cigarette smoking and SHS among conscripts in Taiwan and compare the result of a questionnaire survey with the conscripts’ urine test result. Additionally, the effect of SHS on the UC concentration was explored.

## Methods

### Study Population and Design

**Figure 1:**
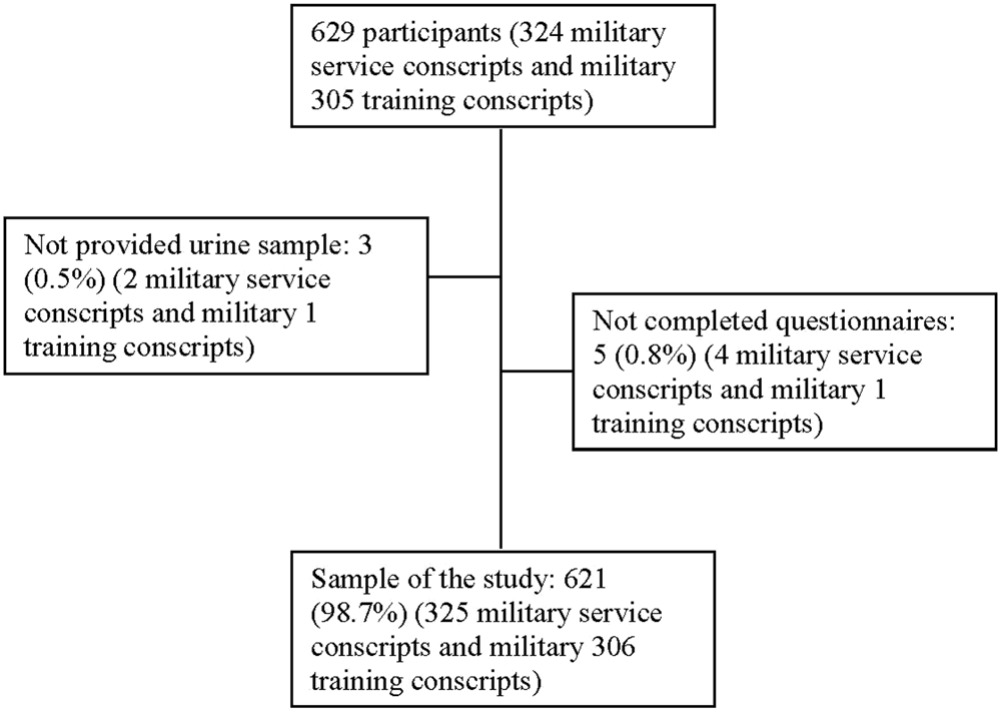
Flow chart of the study.

A cross-sectional study was adopted to examine conscripts who were receiving training in recruit training centres. Two recruit training centres were targeted through purposive sampling, from which two companies were selected using random sampling. In this study, we included male conscripts who were aged at least 18 years old and were being trained at a new training regiment in Taichung city, Taiwan. Two participants were excluded because they were receiving nicotine replacement therapy (NRT), and eight participants with incomplete data were excluded from the final analysis. In total, 621 conscripts were included in the data analysis. After the conscripts were fully informed of the research content, objectives, and the rights of participants, a total of 629 willing participants were recruited (comprising 324 military service conscripts and 305 basic military training conscripts). Figure 1 shows that of the 629 enrolled participants, three did not provide a urine sample and five did not complete the questionnaire. The response rate was 98.7%. Written informed consent was obtained from all participants. The study design and protocols were reviewed and approved by the Institutional Review Board of the Tri-Service General Hospital, National Defense Medical Center and performed according to the guidelines of the Declaration of Helsinki (2–103–05–012).

### Procedure

The data were collected from July 2014 to December 2014 by administering both the questionnaire survey and urine test on the penultimate day of the recruit training. The conscripts were asked to first complete a self-reporting questionnaire and then a urine test.

The urine specimens were analysed within one week after collection using enzyme-linked immunosorbent assay (ELISA) kits (Calbiotech Co., Spring Valley, CA, USA). Cotinine concentrations were measured following the manufacturer’s instructions^13^. The urine samples were stored at −20 °C. Before analysing, urine specimens were brought to room temperature (18–26 °C). In this assay, 10 μl of standards, controls, and specimens were added to the selected wells in duplicate. One hundred microliters of the enzyme conjugation solution was added into each well. The plates were shaken for 10–30 seconds to ensure proper mixing. After 60 minutes of incubation at room temperature (18–26 °C) in the dark, the wells were washed 6 times with 300 μl of distilled water. Then, the wells were inverted and vigorously slapped dry on absorbent paper and 100 μl of substrate reagents were added. After incubating for 30 minutes at room temperature, 100 μl of stop solution was added to each well, and the plate was gently shaken to mix the solution. The limit of detection (LOD) of this assay was 1.0 ng/ml. The coefficient of variation of the assay was within 10% during the period of sample analysis.

### Measurement

The questionnaire used in this study, which was comprised information regarding demographics, smoking status, smoking quantity, nicotine dependence level, and SHS exposure, was designed according to previous investigations^6–10,14^. The question for smoking status was “Do you currently have the habit of smoking?” with the answers of *yes* and *no*. Those who answered *yes* were regarded as smokers, while those who answered *no* were nonsmokers. The question for smoking quantity was “On average, how many cigarettes have you smoked per day in the past 30 days?” For SHS exposure, the question was “Has anyone smoked near you in the past 30 days?”^14^ with the answers of *yes* and *no*. The nicotine dependence level was measured using the Chinese version of the Fagerstrom Test for Nicotine Dependence (FTND), which has six questions^15^.

The UC concentration was adopted as the biomarker. The rate of nicotine metabolism varies among people of different races. This study referred to previous studies that have been conducted in South Korea, and smokers were identified as those with UC levels of ≥100 ng/mL^10,16^.

### Statistical Analysis

Statistical analysis was performed using IBM SPSS Statistics 22.0 software. The results from the military service participants and the basic military training participants were compared. The variables of age, educational degree, smoking quantity, and nicotine dependence level were described by numerical values, namely frequency, percentage, mean, and standard deviation. In addition, the chi-squared (χ^2^) test and independent t-test were performed to compare the demographic data of the military service conscripts with those of the basic military training conscripts. The conscripts’ self-reported smoking behaviours and urine test results were compared using the kappa coefficient, sensitivity, and specificity. Additionally, generalized estimating equations (GEE) analysis was used to compare the concordance of self-reported and cotinine-validated smoking status and to adjust for covariates. In the equation, the dependent variable was smoking status (smoker and nonsmoker). Independent variables were age, educational attainment level, and SHS exposure. The relationship between the SHS exposure level and UC concentration for the military service conscripts was compared with that for the basic military training conscripts by the Mann-Whitney U test.

## Results

### Characteristics of Conscripts

The conscripts in this study consisted of 318 military service conscripts and 303 basic military training conscripts (Table 1). All the conscripts were male. The conscripts averaged 20.9 years of age, with the military service conscripts being significantly older than the basic military training conscripts (22.5 and 19.1 years old, respectively; *P*-value < 0.001). The educational attainment levels of the two groups differed significantly. Approximately 91% of the military service conscripts graduated from a junior college or above, whereas 94.4% of the basic military training conscripts graduated from a senior high school or below. The self-reported smoking rates of the conscripts was 30.9%, which was significantly higher in the basic military training conscripts (39.3%) than in the military service conscripts (23.0%). In addition, the basic military training conscripts were exposed to a significantly higher level of SHS than were the military service conscripts (47.9% and 11.3%, respectively; *P*-value < 0.001). The FTND scores for the two groups differed non-significantly.

**Table 1:** Characteristics of conscripts.

Type of military services	Overall N = 621	Military service conscript n = 318	Basic military training conscript n = 303	*P*-value^a^
Age (Mean ± SD)	20.9 ± 1.9	22.5 ± 1.0	19.1 ± 0.8	<0.001
Educational attainment level				<0.001
Senior high school and below	281 (50.1)	27 (9.2)	254 (94.4)	
Junior college and above	280 (49.9)	265 (90.8)	15 (5.6)	
Self-reported smoking status				<0.001
Yes	192 (30.9)	73 (23.0)	119 (39.3)	
No	429 (69.1)	245 (77.0)	184 (60.7)	
SHS exposure				<0.001
Yes	181 (29.1)	36(11.3)	145(47.9)	
No	440 (70.9)	282(88.7)	158(52.1)	
Smoking quantity (Mean ± SD)^b^	10.1 ± 5.8	10.8 ± 6.3	9.8 ± 5.4	0.275
Score of nicotine dependence (Mean ± SD)^bc^	3.0 ± 2.2	2.6 ± 2.1	3.2 ± 2.2	0.101

^a^The results of military service and basic military training conscripts were compared by conducting the χ2 test and independent t-test.

^b^Only self-reported smokers were analysed.

^c^Nicotine dependence was measured using the FTND.

### Concordance of Self-reported and Cotinine-validated Smoking Status among Conscripts

Among all the conscripts, 29.6% (n = 184) were self-reported smokers whose UC concentrations were ≥100 ng/mL, and 66.8% (n = 415) were self-reported nonsmokers whose UC concentrations were <100 ng/mL. The overall agreement rate was 96.4%, and the kappa coefficient was 0.918 (*P*-value < 0.001). A total of 302 military service conscripts demonstrated consistent self-reported and cotinine-validated smoking statuses. The agreement rate was 94.9%, and the kappa coefficient was 0.862 (*P*-value < 0.001). Among the basic military training conscripts, 297 participants (98.0%) had consistent self-reported and cotinine-validated smoking statuses, with a kappa coefficient value of 0.958 (*P*-value < 0.001; Table 2). Results from the GEE model (Table 3) further confirmed that after the covariates were controlled for, the frequency of smoking behavior between self-reported and cotinine-validated smoking behavior did not differ significantly among all the conscripts (*P*-value = 0.107), military service conscripts (*P*-value = 0.655), and basic military training conscripts (*P*-value = 0.108).

**Table 2:** Concordance of self-reported and cotinine-validated smoking status among conscripts.

Type of military services	Self-reported Smoking Status	Cotinine-validated Smoking Status		
		≥100 ng/mL n (%)	<100 ng/mL n (%)	Total
Overall	Smoker	184 (29.6)	8 (1.3)	192 (30.9)
	Nonsmoker	14 (2.3)	415 (66.8)	429 (69.1)
	Total	198 (31.9)	423 (68.1)	621 (100.0)
		Kappa coefficient = 0.918^***^		
Military service conscript	Smoker	68 (21.4)	5 (1.6)	73 (23.0)
	Nonsmoker	11 (3.5)	234 (73.5)	245 (77.0)
	Total	79 (24.9)	239 (75.1)	318 (100.0)
		Kappa coefficient = 0.862^***^		
Basic military training conscript	Smoker	116 (38.3)	3 (1.0)	119 (39.3)
	Nonsmoker	3 (1.0)	181 (59.7)	184 (60.7)
	Total	119(39.3)	184 (60.7)	303 (100.0)
		Kappa coefficient = 0.958^***^		

^***^ *P*-value < 0.001.

**Table 3:** Results of GEE for self-reported and cotinine-validated smoking status.

Parameter	Overall	Military service conscript	Basic military training conscript			
	OR (95%CI)	*P*-value	OR (95%CI)	*P*-value	OR (95%CI)	*P*-value
Smoking status		0.107		0.655		0.108
Cotinine-validated	1		1		1	
Self-reported	0.931 (0.854~1.016)		0.979 (0.890~1.076)		0.883 (0.758~1.028)	
Age	0.766 (0.640~0.917)	0.004	0.459 (0.307~0.686)	<0.001	0.691 (0.489~0.976)	0.036
Educational attainment level		0.143		0.442		0.188
Senior high school and below	1		1		1	
Junior college and above	1.678 (0.840~3.354)		1.737 (0.425~7.106)		0.592 (0.272~1.292)	
SHS exposure		<0.001		<0.001		0.004
No	1		1		1	
Yes	5.466 (3.606~8.285)		9.066 (5.117~16.062)		3.111 (1.443~6.707)	

GEE = generalized estimating equations.

The sensitivity of self-reported smoking status for all the conscripts was 92.9%, indicating that 92.9% of the conscripts who were identified as smokers by the urine test also reported themselves as smokers. The sensitivity of the self-reported smoking status of the basic military training conscripts was higher than that of the military service conscripts (97.5% and 86.1%, respectively; *P*-value = 0.002). The overall specificity for the conscripts was 98.1%. Although the specificity for the military training soldiers was slightly higher than that for the military service conscripts (98.4% and 97.9%, respectively), the difference was nonsignificant (Table 4).

**Table 4:** Sensitivity and specificity of self-reported smoking status.

	Overall		Military service conscript		Basic military training conscript	
	%	95% CI	%	95% CI	%	95% CI
Sensitivity	92.9	88.5~95.7	86.1	76.8~92.0	97.5	92.9~99.1
Specificity	98.1	96.3~99.0	97.9	95.2~99.1	98.4	95.3~99.4

CI = confidence interval.

### Effect of SHS Exposure on UC Levels

Regardless of the type of military service, the UC levels of the self-reported smokers were higher than those of the self-reported nonsmokers. For the self-reported smokers, the SHS exposure did not affect their UC levels. However, among the self-reported nonsmokers, the military service conscripts who had been exposed to SHS attained higher UC median levels than did those who had not been exposed to SHS (4.4 ng/mL and 2.5 ng/mL, respectively, *P*-value = 0.023) (Table 5).

**Table 5:** The UC levels of conscripts who self-reported their smoking behaviour and SHS exposure^a^.

Type of military services	Self-reported Smoker				Self-reported Nonsmoker				*P*-value^c^
	Total	SHS Exposure	SHS Unexposed	*P*-value^b^	Total	SHS Exposure	SHS Unexposed	*P*-value^b^	
Overall	407.2	408.8	399.6	0.318	2.6	2.7	2.6	0.874	<0.001
Military service conscript	418.9	424.7	418.9	0.700	2.6	4.4	2.5	0.023	<0.001
Basic military training conscript	396.5	407.2	306.9	0.063	2.7	2.6	2.8	0.215	<0.001

^a^Median level of urinary cotinine ng/mL; ^b^ *p*-value was examined by the Mann-Whitney U test; ^c^compared self-reported smoker and nonsmoker by the Mann-Whitney U test; SHS = second-hand smoke.

## Discussion

This study was the first in Taiwan to verify the self-reported smoking behaviour of conscripts. The results showed that, although self-reporting surveys typically underestimate the smoking rate of the respondents, such survey outcomes are still highly consistent with those determined through a urinary test. Thus, self-reporting surveys can serve as an effective tool for assessing smoking behaviour. In this study, the self-reported smoking rate of the conscripts was 30.9%, which was lower than the smoking rate determined using the UC concentration test (i.e., 31.9%). Additionally, for the military service conscripts, the self-reported smoking rate was lower than that determined using the UC concentration test (23.0% and 24.9%, respectively). However, for the basic military training conscripts, both the self-reporting survey and UC concentration test yielded the same smoking rate (39.3%). Some studies on conscripts in Taiwan found that the prevalence of smoking was 51.3% to 52.6%^17–19^. The smoking rate was higher than that noted in our study. This may be due to the different distribution of education level among conscripts. The percentage of conscripts graduating from a senior high school or below in our study was 50%, which was lower than that in prior studies (74%)^17–19^. However, the smoking rate of conscripts was higher than that of males of similar age (16.1%) in the 2014 Adult Smoking Behaviour Survey in Taiwan^20^. The systematic literature review conducted by previous studies has reported that self-reporting data may underestimate smoking rates^5^. A Canadian study targeting the general public found that the self-reported smoking rate was 0.3% lower than the smoking rate determined using a UC concentration test; however, the extent of underestimation was nonsignificant, indicating the effectiveness of self-reporting assessments^6^. The US National Health and Nutrition Examination Survey (NHANES) also asserted that self-reporting methods can be used to accurately measure smoking behavior^21,22^. We can conclude that using a UC concentration test to estimate smoking behaviour is relatively objective compared with a self-reporting survey. The present study confirmed that the conscripts’ self-reported results and cotinine-validated results demonstrated a high consistency level (i.e., the overall kappa coefficient value was 0.918; the kappa coefficient values for the military service and basic military training conscripts were 0.862 and 0.958, respectively). This indicates that a low-cost questionnaire survey which is applicable to large samples can effectively determine the smoking status of conscripts.

The sensitivity and specificity of the self-reported smoking status of all the conscripts were 92.9% and 98.1%, respectively, which aligns with the results of the Canadian study (in which the sensitivity and specificity were 91.6% and 98.3%, respectively)^6^. Furthermore, the sensitivity of the military service conscripts was significantly lower than that of the basic military training conscripts (86.1% and 97.5%, respectively), which might be attributable to the fact that the data collection occurred on the penultimate day of recruit training. After recruit training, basic military training conscripts can leave the military, while military service conscripts are reassigned to various military units to continue their service. Therefore, military service conscripts may conceal their smoking behaviour to meet the military officials’ expectations. Although no previous studies on the military can be compared with this study, similar results have been reported in studies on patients who had smoking-related diseases. Those patients also concealed their smoking behaviour to meet the physicians’ expectations^9,23–25^.

In our study, the prevalence of SHS exposure among conscripts was 29.1%. Moreover, the basic military training conscripts had a higher prevalence of SHS exposure than the military service conscripts (39.3% vs. 23.0%). A study executed by Seo *et al*.^26^ found that the self-reported prevalence of SHS exposure in 7 days among college students was 45%. The 2014 Adult Smoking Behaviour Survey in Taiwan revealed that 64.6% of men aged 18 to 24 experienced SHS^20^. The rate of SHS exposure among conscripts in our study was lower than that among college students and similar age groups. This may be due to the fact that the data were collected on the penultimate day of the recruit training in our study, and conscripts were exposed to more smoking-restricted environments than college students and other young males. The present study further analysed the effect of SHS exposure on the UC concentrations for the two participant groups. Among the self-reported nonsmokers, the military service conscripts exposed to SHS demonstrated significantly higher UC concentrations than did those who had not been exposed to SHS (4.4 ng/mL and 2.5 ng/mL, respectively; *p* = 0.023). Aurrekoetxea *et al*.^6^ also reported that the UC median levels of pregnant nonsmokers who had been exposed to SHS was higher than that of those who had not been exposed to SHS (7.6 ng/mL and <4 ng/mL, respectively). SHS exposure increased UC concentrations by 1.9 ng/mL among military service conscripts who did not smoke. Previous studies have suggested that a UC concentration of >20 ng/mL can be a baseline for assessing SHS exposure^25^. However, in this study, the UC median levels of the self-reported nonsmokers did not reach the baseline. This may be because the Taiwanese military has implemented smoking prohibition policies, which only permit soldiers to smoke in the smoking zone during the recruit training period. Thus, the effect of SHS exposure on the nonsmokers was minimal.

Although this study verified the accuracy of the conscripts’ self-reported smoking behaviour, several research limitations remained. First, human genes affect the rate of nicotine metabolism and further influence the UC concentrations^27–29^. Therefore, the baseline of UC level affects the accuracy of smoking status assessment. Although previous studies have defined the baseline UC level for smokers as 50 ng/mL^6,30^, the present study considered the effect of genes and adopted 100 ng/mL as the baseline by referring to Korean studies. Second, diet also influences UC concentrations in a range from 0.6 to 6.2 ng/mL^31^. The present study did not collect data related to diet; however, during the training period, the meals were all prepared by the military. Thus, differences in diet may have exerted minimal influence on the UC concentrations of the conscripts in this study. Third, this study conducted a questionnaire survey to assess the SHS exposure level over a period of 30 days. However, the half-life of nicotine metabolism was 16 to 20 hours; hence, the observed UC concentrations may be unable to reflect the influence of SHS exposure. Nonetheless, because all the conscripts experienced the same environment during recruit training, using the UC concentrations to represent the SHS exposure level was still feasible. Finally, this study is limited because our sample consists only of male conscripts who were being trained at a new training regiment; hence, the accuracy of self-reporting of smoking behaviour in the study may not be representative of that of the entire military population. In addition, because the study did not include a control group of the general population, the results should be carefully generalized to soldiers in other service types. Future studies should extend assessments to the entire military population.

Despite the above-mentioned limitations, this study was the first in Taiwan to objectively verify conscripts’ self-reported smoking behaviour using UC concentrations as a biomarker. In addition, because the questionnaire survey and urine test were performed on the same day, the error of incorrect categorization of smoking behaviour that arises from different data collection periods can be reduced.

## Conclusion

This study found that the smoking behaviour self-reported by the participants in the questionnaire survey was highly consistent with that determined using the UC concentration test. Subsequent studies can also conduct questionnaire surveys to monitor the smoking behaviour of conscripts. Moreover, future scholars are recommended to investigate the smoking behaviour of soldiers at various hierarchical positions to confirm the accuracy of their self-reported smoking behaviour. Furthermore, future studies are recommended to obtain further information on the factors associated with inaccurate self-reported of smoking status, such as sociodemographic background, knowledge of tobacco hazards, and tobacco cessation history.
